# Effect of Aqueous Extract from* Descurainia sophia* (L.) Webb ex Prantl on Ventricular Remodeling in Chronic Heart Failure Rats

**DOI:** 10.1155/2018/1904081

**Published:** 2018-06-13

**Authors:** Ying Luo, Zhiqiang Sun, Pengfei Hu, Yuanhong Wu, Weiwei Yu, Shuwei Huang

**Affiliations:** The Second Clinical Medicine College of Zhejiang Chinese Medical University, Hangzhou, Zhejiang 310005, China

## Abstract

**Objective:**

* Descurainia sophia* (L.) Webb ex Prantl (DS) is a traditional Chinese medicine. Our current study was to evaluate the effect of DS on ventricular remodeling in chronic heart failure (HF) rats and its underlying mechanism.

**Methods:**

The rat chronic heart failure model induced by suprarenal abdominal aortic coarctation surgery. The survival rats were randomly divided into 3 groups: the sham group (n=6), the HF group (n=6), and the HF+DS group (n=6). After 3 months of drug intervention, we examined the effects of DS by Sirius Red staining, electron microscopy, echocardiography, hemodynamic measurement, and TUNEL and explored the underlying mechanism by Western blotting.

**Results:**

We found that rats treated with DS showed improved cardiac function and less tissue damage compared to untreated group. Additionally, DS could reduce the cardiomyocytes apoptosis, decrease the ratio of Bax/bcl-2 and Caspase-3 expression, and enhance the phosphorylation of Akt protein expression.

**Conclusion:**

Our study suggested that rats treated with DS after suprarenal abdominal aortic coarctation surgery showed attenuated cardiac fibrosis and apoptosis, and the protective effect may be correlated with the activation of PI3k/Akt/mTOR dependent manner.

## 1. Introduction

Chronic heart failure is the end-stage manifestation of all cardiovascular diseases [1]. The prevalence of cardiovascular disease in China is increasing rapidly, and chronic heart failure has become an important public health problem in the cardiovascular field. At present, the incidence of heart failure in China is 0.9%; mortality continues to increase with age [2]. Ventricular remodeling is the core pathogenesis of heart failure development [3]; it is also the major reason for the morbidity, progression, and mortality of heart failure [4, 5]. DS is a member of the family Brassicaceae, which is widespread in Asia, Europe, northern Africa, and North America. The seeds of DS have been used to promote urination, relieve cough and asthma, and enhance cardiac function for a long time [6]. Previous basic research had shown that this medicine has the effect of improving cardiac function [7], but its mechanism is unclear. This study was to explore the cardiac protective effect of DS and its underlying mechanisms by using a rat chronic heart failure model induced by suprarenal abdominal aortic coarctation surgery.

## 2. Materials and Methods

### 2.1. Animal Model and Study Design

In this study, male Sprague-Dawley rats weighing ~250 g were used. The chronic heart failure procedure was performed as per the following description. Firstly, the rats were anesthetized with 10% pentobarbital (50 mg/kg intraperitoneal). A subxiphoid left para-median abdominal incision was made to open the abdominal cavity; then, the abdominal aorta was bluntly dissected. Subsequently, a syringe with a size 8 needle from which the tip of the needle was removed was placed parallel to the abdominal aorta and ligated with line 4. Afterwards, the tip of the needle was gently drawn out so that the area of the abdominal aorta decreased to 50 to 60% of the original size. Penicillin powder was sprinkled, and the abdomen was closed; layer-to-layer suturing was then performed. The sham group underwent all the same procedures except suprarenal abdominal aortic coarctation. Chronic heart failure rats were taken and randomly allocated into either the sham group, the HF group, or the HF+DS group. The HF+DS group was given DS (10 g/kg.d intragastric); the sham and HF groups were given the same volume of normal saline used for DS dissolution. All animals were fed under the same condition for 3 months.

### 2.2. Echocardiographic Measurements

Echocardiographic dates including the left ventricular inner diameter at the end of systole(LVESD), the left ventricular inner diameter at the end of diastole(LVEDD), left ventricular ejection fraction [LVEF=(LVEDD^3^-LVESD^3^)/LVEDD^3^], and left ventricular fractional shortening [FS=(LVEDD-LVESD)/LVEDD] were obtained by using a 21-MHZ phased-array transducer (Vevo2100)[8]. Measurement and analysis were completed by the same person, who was blind to the condition.

### 2.3. Hemodynamic Measurements

After echocardiographic measurements, a fluid-filled polyethylene microcatheter (P50) was inserted into the right common carotid artery by connecting to a pressure transducer (TRI 21. Letica Scientific Instruments) to record the hemodynamic parameters of Power lab [ML750 (AD instrument)]. After the microcatheter entered the left ventricle, the left ventricle systolic pressure (LVSP), the maximum positive and negative values of dP/dt (±dP/dt max) and the left ventricular end diastolic pressure (LVEDP) were measured when hemodynamic was stable [8].

### 2.4. Cardiac Fibrosis Assessment

Left ventricular myocardial specimens were taken and fixed in 4% formaldehyde for 24 h. After material extraction, dehydration, and paraffin embedding, 4 *μ*m thickness sections were taken along the long axis of the left ventricle. After HE staining, histomorphological changes of the myocardium were observed under a light microscope [9].

### 2.5. Transmission Electron Microscopy

Left ventricular myocardium was removed and fixed with 2.5% glutaraldehyde overnight at 4°C, then washed three times with phosphate buffer, and fixed with 1% osmium tetroxide for 2 hr. Three ultrathin sections were obtained via an ultra-small (Leica, Solms, Germany) standard procedure on an uncoated copper grid and stained with 0.2% lead citrate/1% uranyl acetate. Images were recorded through a transmission electron microscope (JOEL-1400EX, Tokyo, Japan) and observed at a magnification of 20000x.

### 2.6. Western Blot Analysis

The rat left ventricular myocardium tissue samples were crushed in liquid nitrogen, added to the lysate for homogenization, and centrifuged at 13000 rpm for 10 minutes, and the supernatant was assayed for protein concentration by BCA method and stored at −20°C after denaturation. Samples (30 *μ*g/lane) were electrophoresed on 12% SDS-PAGE and transferred to PVDF membranes. The membranes were blocked with 3% BSA for 2 hr at room temperature and then were incubated with the primary antibodies (1:1000 dilution) overnight at 4°C. After being washed three times with TBST, the membranes were incubated with secondary antibody (1:5000 dilution) at room temperature for 2 hr. After being washed three times with TBST, the enhanced chemiluminescence (ECL) reagent was developed. Protein expression levels were observed via a chemi-doc image analyzer (FluorChemo M FM0488) and analyzed with ImageJ. GAPDH (1:1000 dilution) was used as an internal reference, and the ratio results indicated the relative protein content.

### 2.7. Statistical Analysis

All data were expressed as the mean ± SD. The SPSS 22.0 software was performed with a one-way analysis of variance (ANOVA) for multiple comparisons. The values of* P*<0.05 was considered statistically significant.

## 3. Results

### 3.1. Descurainia sophia Improves Morphological Alteration of Myocardial Fibers

**Figure 1:**
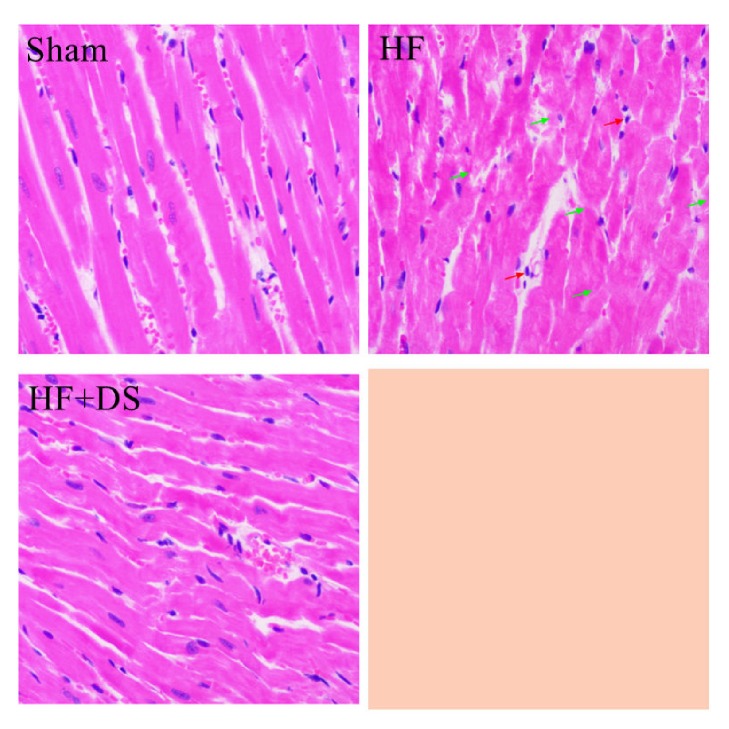
DS could improve HF-induced morphology alterations in cardiomyocytes examined by HE staining. Red arrow represents inflammatory cells, and green arrow represents the broken and thicker myocardial fiber.

In the sham group, myocardial fibers were arranged regularly without breaking, and their structures were normal. In the HF group, myocardial fibers were broken and hypertrophied, with inflammatory cells in the interstitial space, but treatment with DS attenuated these effects (Figure 1).

### 3.2. Descurainia sophia Improved Cardiac Function in CHF Rats

**Figure 2:**
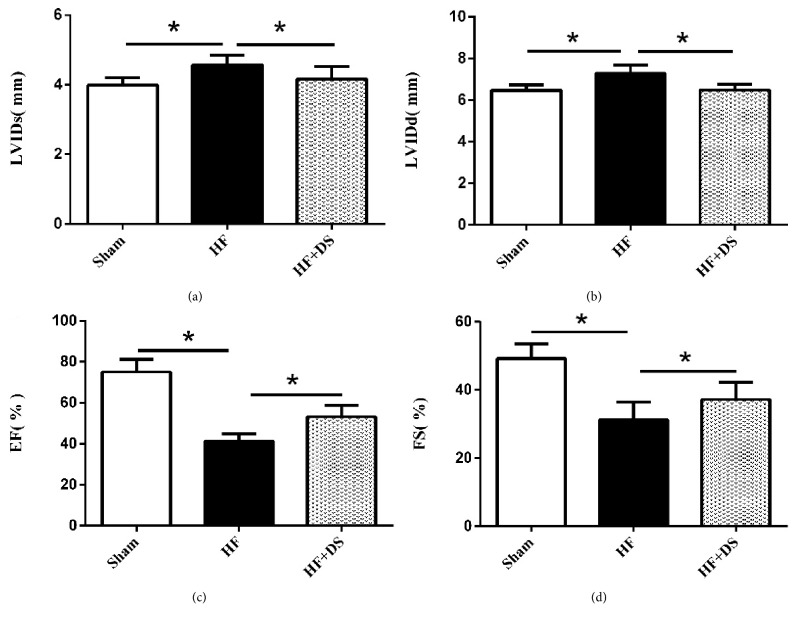
Echocardiographic data showed that rats treated with DS had higher EF (%) and FS (%) and lower LVIDs and LVIDd compared to untreated group. This result indicated that DS could improve cardiac function in heart failure rats. At least 6 consecutive cardiac cycles were measured for each rat. Values were mean ± SD. *∗P <*0.05 versus HF group.

**Figure 3:**
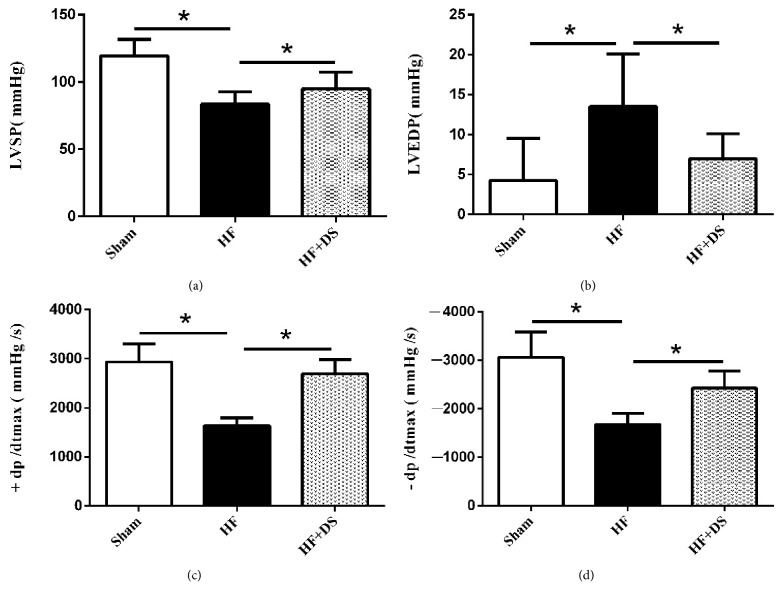
Hemodynamic assessments showed rats treated with DS had lower LVEDP and higher LVSP and ±dp/dtmax compared to untreated group. This result showed that DS could improve myocardial contractility and relaxation in chronic heart failure rats induced by suprarenal abdominal aortic coarctation surgery. Values were mean ± SD. *∗P <*0.05 versus HF group.

Three months after the operations, compared with the HF group, the HF+DS group had significantly lower LVIDd and LVIDs and higher LVEF and FS (Figure 2). Hemodynamic measurement showed increased LVSP and decreased LVIDd and ±dp/dt in DS-treated rats (Figure 3). Details are shown in Table 1.

### 3.3. Descurainia sophia Improved Myocardial Ultrastructure

**Figure 4:**
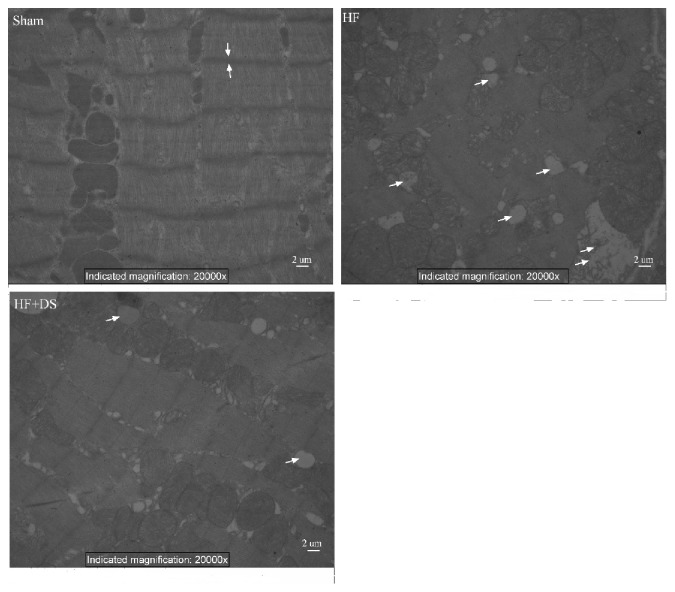
DS suppresses morphologic alterations in cardiomyocytes in HF rats examined by an electron microscope.

In the sham group, the myocardial fibers were properly organized, the z line was clear and uniform, and the mitochondrial crest was dense and orderly. In the HF group, the myocardial fibers were disorganized, and the z line thickness disappeared and even broke. The mitochondrial morphology was different, the membrane structure was unclear, and the crest was disordered, fused, or even disappeared; mitochondria vacuolization and swelling were observed. In the HF+DS group, these pathologic changes could be reversed (Figure 4).

### 3.4. Descurainia sophia Attenuated Cardiomyocyte Apoptosis

**Figure 5:**
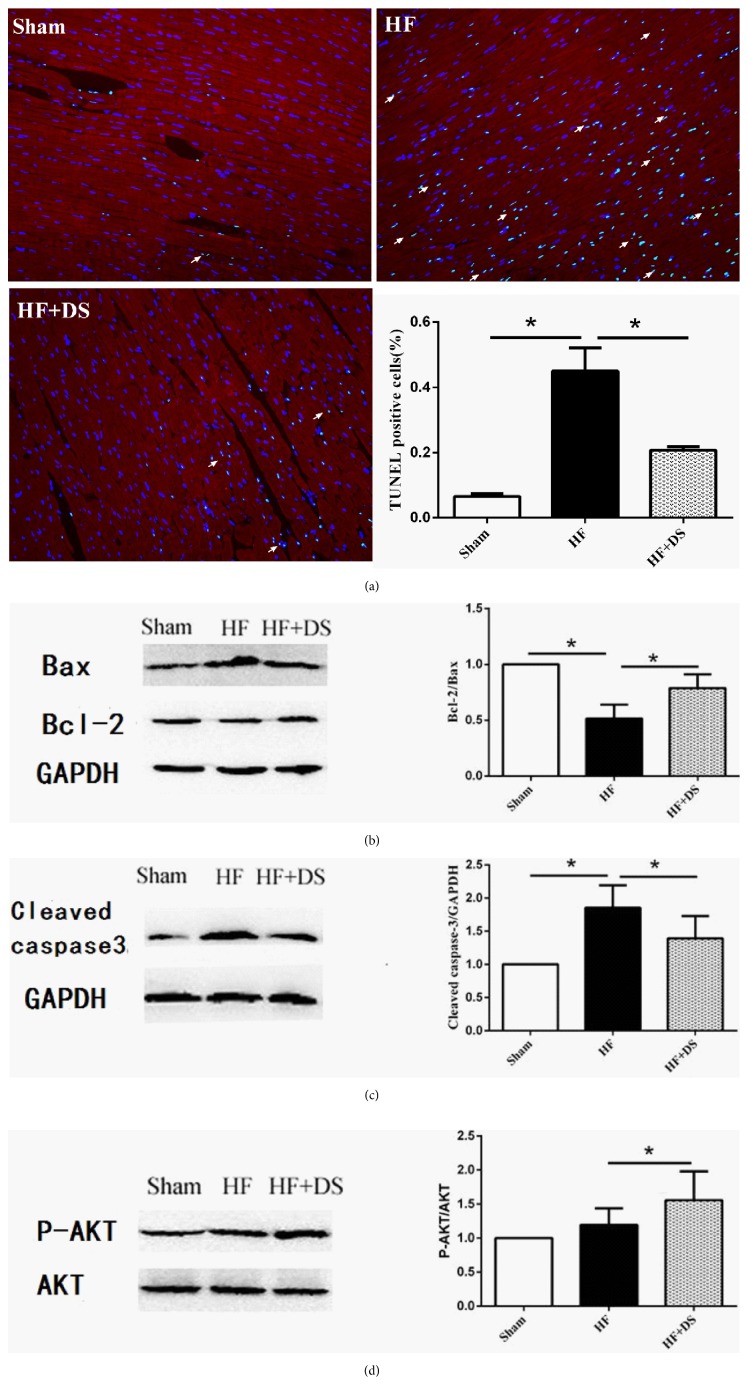
(a) Double staining of TUNEL and cTnI in myocardial tissue (scale bar: 20 *μ*m). Green indicated the TUNEL-positive cell nucleus, and red indicated the cTnI signals. TUNEL-positive cells were counted and averaged in four randomly selected high-power fields around the border zone for each section. The results showed that DS decreased myocardial apoptosis compared with the HF group. Values were mean ± SD. *∗P <*0.05 versus HF group. (b), (c), (d) DS could decrease the ratio of Bax/bcl-2 and cleaved-Caspase-3 protein expression and enhance the p-Akt protein expression. Values were mean ± SD. *∗P <*0.05 versus HF group.

Cardiomyocyte apoptosis is a potential mechanism in the development of heart failure. TUNEL showed that DS could reduce the cardiomyocytes apoptosis (Figure 5(a)). Compared with the sham group, the proapoptotic factor Bax expression was significantly increased while the overexpression of antiapoptotic factor Bcl-2 and Caspase-3 was observed in the HF group. By contract, the HF+DS group decreased the ratio of Bax/bcl-2 and Caspase-3 and enhanced phosphorylation of Akt protein expression (Figures 5(b), 5(c), and 5(d)).

## 4. Discussion

Adverse remodeling after pressure overload is characteristic of heart failure; it is the result of the interaction of hemodynamic abnormalities and neuroendocrine disorders [10]. Currently, there are limited treatments for improving ventricular remodeling in heart failure patients. However, with the progression of modern technology, herbal compound extracts are increasing successfully in clinical practice. Previous pharmacological studies have shown that* Descurainia sophia* (L.) Webb ex Prantl had excellent performance in “harmful fluid retention in the upper jiao” model [11], a Chinese medicine syndrome model characterized by cough, asthma, chest tightness, and palpitation [12]. However, whether the improvement of ventricular remodeling by* Descurainia sophia* (L.) Webb ex Prantl is associated with inhibiting the cardiomyocyte apoptosis remains to be determined.

In this study, we used a rat chronic heart failure model to evaluate the protective effect of* Descurainia sophia* (L.) Webb ex Prantl on ventricular remodeling and explore the underling mechanisms. Several observations were particularly noteworthy. Firstly, we found that* Descurainia sophia* (L.) Webb ex Prantl improved cardiac function and hemodynamics in chronic heart failure rats induced by suprarenal abdominal aortic coarctation surgery. Secondly, cardiac fibrosis was attenuated with* Descurainia sophia* (L.) Webb ex Prantl treatment. Finally,* Descurainia sophia* (L.) Webb ex Prantl could reduce the cardiomyocyte apoptosis.

Cell apoptosis is an active and programmed death process under physiological or pathological conditions and is an important self-stabilizing mechanism of the body [13]. The decrease of myocardial cells induced by apoptosis is one of the important mechanisms for the development of compensatory myocardial hypertrophy under a long period of hemodynamic overload [14]. It has been reported that excessive cardiomyocyte apoptosis after pressure overload is related with cardiac dysfunction [15]. A large number of studies have shown that the bcl-2 family and the caspase family play an extremely important regulatory role in the cell apoptosis transduction pathway, especially in the mitochondrial pathway. Bcl-2 and bax protein are located upstream from the mitochondria and are important regulatory genes for mitochondrial membrane permeability changes. Their overexpression can control the release of the upstream cyt-c and the activation of downstream caspase-3 protease and mediate cell apoptosis [16]. Our study suggested that* Descurainia sophia* (L.) Webb ex Prantl protects cardiomyocyte apoptosis at least partially through the Bcl-2/Bax mediated mitochondrial apoptosis signaling pathway. PI3K/Akt/mTOR dependent manner as a classical signaling pathway had been shown to inhibit cardiomyocyte apoptosis and protect myocardium [17]. Our study revealed that the level of phosphor-Akt was elevated and the cardiomyocytes apoptosis was reduced compared to untreated group, so the cardioprotective effect of* Descurainia sophia* (L.) Webb ex Prantl may be correlated with the activation of the PI3K/Akt/mTOR dependent manner.

Several limitations in the present study are as follows. Firstly, traditional Chinese medicine is characterized by its complex composition and complicated mechanism. The absence of appropriate research methods leads to the fact that the mechanisms of most traditional Chinese medicine are difficult to clarify. Previous studies on the relationship between chemical composition and therapeutic effect are based on either one type of compound, flavonoids [18], alkaloids, triterpenoids [19], or a total extract [20]. None of the above compounds could fully reflect the characteristics of every type of compound in traditional Chinese medicine and its contributions to the overall efficacy. Secondly, there is no traditional Chinese medicine that is proven to improve ventricular remodeling in clinical guidance; thus, we did not establish a positive control group. Finally, we did not further clarify the mechanism of apoptosis at the cellular level, and this will be our next area of study.

In conclusions,* Descurainia sophia* (L.) Webb ex Prantl could significantly improve remodeling and cardiac function in chronic heart failure rats induced by pressure overload with suprarenal abdominal aortic coarctation surgery. The underling mechanism may be correlated with the attenuation of cardiomyocyte apoptosis mediated by regulating the balance between Bax and Bcl-2, blocking caspase cascades with the activation of PI3k/Akt/mTOR dependent manner. However,* Descurainia sophia* (L.) Webb ex Prantl may also inhibit cardiomyocyte apoptosis through other means; these possible mechanisms require further investigation. By* Descurainia sophia* (L.) Webb ex Prantl as a traditional Chinese medicine, our current study indicated a new perspective in the molecular mechanisms for improving cardiac function in the chronic heart failure model, and it had a protective effect in the chronic heart failure rats induced by suprarenal abdominal aortic coarctation surgery, making it evidence-based both fundamentally and clinically.

## Figures and Tables

**Table 1:** Echocardiographic and hemodynamic assessments of LV function.

	**Sham**	**HF**	**HF+DS**
**LVIDs (mm)**	3.98±0.22*∗*	4.57±0.28	4.16±0.37*∗*
**LVIDd (mm)**	6.47±0.26*∗*	7.27±0.42	6.47±0.28*∗*
**EF (**%**)**	75.00±6.11*∗*	41.25±3.58	53.21±5.54*∗*
**FS (**%**)**	49.21±4.32*∗*	31.27±5.14	37.20±5.04*∗*
**LVSP (mmHg)**	119.59±12.06*∗*	83.53±9.10	94.84±12.64*∗*
**LVEDP (mmHg)**	-4.25±5.25*∗*	12.52±6.54	6.99±3.12*∗*
**+dp/** **d** **t**_**max**_ ** (mmHg/s)**	2933.91±368.46*∗*	1635.75±159.00	2695.66±286.17*∗*
**-dp/** **d** **t**_**max**_ ** (mmHg/s)**	-3060.26±524.32*∗*	-1679.84±226.68	-2428.02±353.84*∗*

Values were mean ±SD. *∗P<*0.05 versus HF group.

## References

[1] Ponikowski P., Voors Adriaan A., Anker Stefan D. (2016). 2016 ESC Guidelines for the diagnosis and treatment of acute and chronic heart failure: The Task Force for the diagnosis and treatment of acute and chronic heart failure of the European Society of Cardiology (ESC)Developed with the special contribution of the Heart Failure Association (HFA) of the ESC. *European Journal of Heart Failure*.

[2] Yang Y.-N., Ma Y.-T., Liu F. (2010). Incidence and distributing feature of chronic heart failure in adult population of Xinjiang. *Zhonghua Xin Xue Guan Bing Za Zhi*.

[3] Hutchinson K. R., Stewart J. A., Lucchesi P. A. (2010). Extracellular matrix remodeling during the progression of volume overload-induced heart failure. *Journal of Molecular and Cellular Cardiology*.

[4] Jaiswal A., Nguyen V. Q., Carry B. J., le Jemtel T. H. (2016). Pharmacologic and Endovascular Reversal of Left Ventricular Remodeling. *Journal of Cardiac Failure*.

[5] Shah A. M. (2013). Ventricular remodeling in heart failure with preserved ejection fraction. *Current Heart Failure Reports*.

[6] Zhou X.-D., Tang L.-Y., Zhou G.-H., Kou Z.-Z., Wang T., Wang Z.-J. (2014). Advances on Lepidii Semen and Descurainiae Semen. *China journal of Chinese materia medica*.

[7] Zhou N., Sun Y., Zheng X. (2017). A Metabolomics-Based Strategy for the Mechanism Exploration of Traditional Chinese Medicine: Descurainia sophia Seeds Extract and Fractions as a Case Study. *Evidence-Based Complementray and Alternative Medicine*.

[8] Liang T., Zhang Y., Yin S. (2016). Cardio-protecteffect of qiliqiangxin capsule on left ventricular remodeling, dysfunction and apoptosis in heart failure rats after chronic myocardial infarction. *American Journal of Translational Research*.

[9] Lu J., Xiao J., Luo H., Chen H. (2001). Apoptosis in pressure overload-induced heart hypertrophy. *Shengwu Yixue Gongchengxue Zazhi/Journal of Biomedical Engineering*.

[10] Carruth E. D., McCulloch A. D., Omens J. H. (2016). Transmural gradients of myocardial structure and mechanics: Implications for fiber stress and strain in pressure overload. *Progress in Biophysics and Molecular Biology*.

[11] Sun Y. P., Yang S. L., Si Y. P. Effect of Aqueous Extract of the seeds of Descurainia sophia(L.) Webb ex Prantl. On the Rat Model of Harmful Fluid Retention in the Upper Jiao.

[12] Xie W., Ji X. M., Pang Z. X., Wang S. J. (2015). Establishment and evaluation of the rat model of harmful fluid retention in the upper jiao. *World Journal of Integrated Traditional and Western Medicine*.

[13] Meier P., Finch A., Evan G. (2000). Apoptosis in development. *Nature*.

[14] Hang T., Jiang S., Wang C., Xie D., Ren H., Zhuge H. (2007). Apoptosis and expression of uncoupling protein-2 in pressure overload-induced left ventricular hypertrophy. *Acta Cardiologica*.

[15] Liu W., Wang X., Mei Z. (2015). Chronic stress promotes the progression of pressure overload-induced cardiac dysfunction through inducing more apoptosis and fibrosis. *Physiological Research*.

[16] Sun Y., Lin Y., Li H., Liu J., Sheng X., Zhang W. (2012). 2,5-Hexanedione induces human ovarian granulosa cell apoptosis through BCL-2, BAX, and CASPASE-3 signaling pathways. *Archives of Toxicology*.

[17] Chaanine A. H., Hajjar R. J. (2011). AKT signalling in the failing heart. *European Journal of Heart Failure*.

[18] Mao Z., Gan C., Zhu J. (2017). Anti-atherosclerotic activities of flavonoids from the flowers of Helichrysum arenarium L. MOENCH through the pathway of anti-inflammation. *Bioorganic & Medicinal Chemistry Letters*.

[19] Xu H., Yuan Z.-Z., Ma X. (2017). Triterpenoids with antioxidant activities from Myricaria squamosa. *Journal of Asian Natural Products Research*.

[20] Choi J. Y., Hwang C. J., Lee H. P., Kim H. S., Han S.-B., Hong J. T. (2017). Inhibitory effect of ethanol extract of Nannochloropsis oceanica on lipopolysaccharide-induced neuroinflammation, oxidative stress, amyloidogensis and memory impairment. *Oncotarget *.

